# Supplementary material for: Latitudinal patterns in stabilizing density dependence of forest communities
Source: Nature. 2024 Feb 28;627(8004):564–71. doi: 10.1038/s41586-024-07118-4 (PMC10954553; doi:10.1038/s41586-024-07118-4)
Supplement: Supplementary file 1 — This file contains Supplementary Methods and Supplementary Notes. [file 41586_2024_7118_MOESM1_ESM.pdf]

nature portfolio

https://doi.org/10.1038/s41586-024-07118-4

Supplementary information

Latitudinal patterns in stabilizing density dependence of forest communities

In the format provided by the
authors and unedited

nature portfolio

https://doi.org/10.1038/s41586-024-07118-4

Supplementary information

Latitudinal patterns in stabilizing density dependence of forest communities

In the format provided by the authors and unedited

Supplementary methods

Estimating CNDD minus HNDD from our reparameterized model

Assume we fit a (generalized) linear regression of the form

```
M = link(y) ~ a0 + a1*x1 + a2*x2
```

and calculate the estimator of d = a2 – a1 based on this regression. The identical effect d can alternatively be estimated with a regression of the form

```
M’ = link(y) ~ a0’ + a1’*x1 + a2’*(x1 + x2)
```

In the second regression, a0’ and a2’ will be identical to a0 and a2 in the previous regression, while the estimator a1’ will be identical to difference d = a2 – a1. The advantage of fitting the second regression is that p-values and standard errors for d are directly estimated and do not need to be propagated through a2 – a1 via posthoc tests.

To understand why the relationship holds, consider that any model M’ of identical form as M in which the predictors are expressed as a bijective linear combination of the predictors in M will give us the same fit to the data, as well as the same predictions. The only thing that changes when going from M to M’ are the estimated effect sizes.

Specifically, in the second regression M’, the effect for x1 + x2 will incorporate the effect of x1 that is identical to x2, while the predictor x1 will estimate only the part that is not identical to x2. We can check this intuition with a short R simulation, which shows that the claim made above holds exactly:

```
n = 1000
x1 = runif(n)
x2 = runif(n)
y = x1 + 0.5*x2 + rnorm(n, sd = 0.2)

# Regression 1
mod = lm(y ~ x1 + x2)
summary(mod)

# Difference
estimates = coef(mod)
estimates[2] - estimates[3]
```

```
# Posthoc test for SE and significance of the difference
contrast = matrix(c(0, 1, -1), byrow = T, nrow = 1)
summary(multcomp::glht(mod, linfct = contrast))

# Regression 2
summary(lm(y ~ x1 + I(x1 + x2)))
```

Note that in our specific case, where x1 = conspecific density and x2 = heterospecific density, the identity also implies that the estimate for total density in the second regression and the estimate for HNDD in the first regression are identical. The reason is that the effect for total density that is fitted in this case is not to be interpreted as "average density dependence", but as the density dependence of any kind of neighbor. This way it becomes clear that estimate d, which we refer to as 'stabilizing CNDD', estimates "excess density dependence" that is exerted by conspecifics compared to the general community.

Supplementary figures

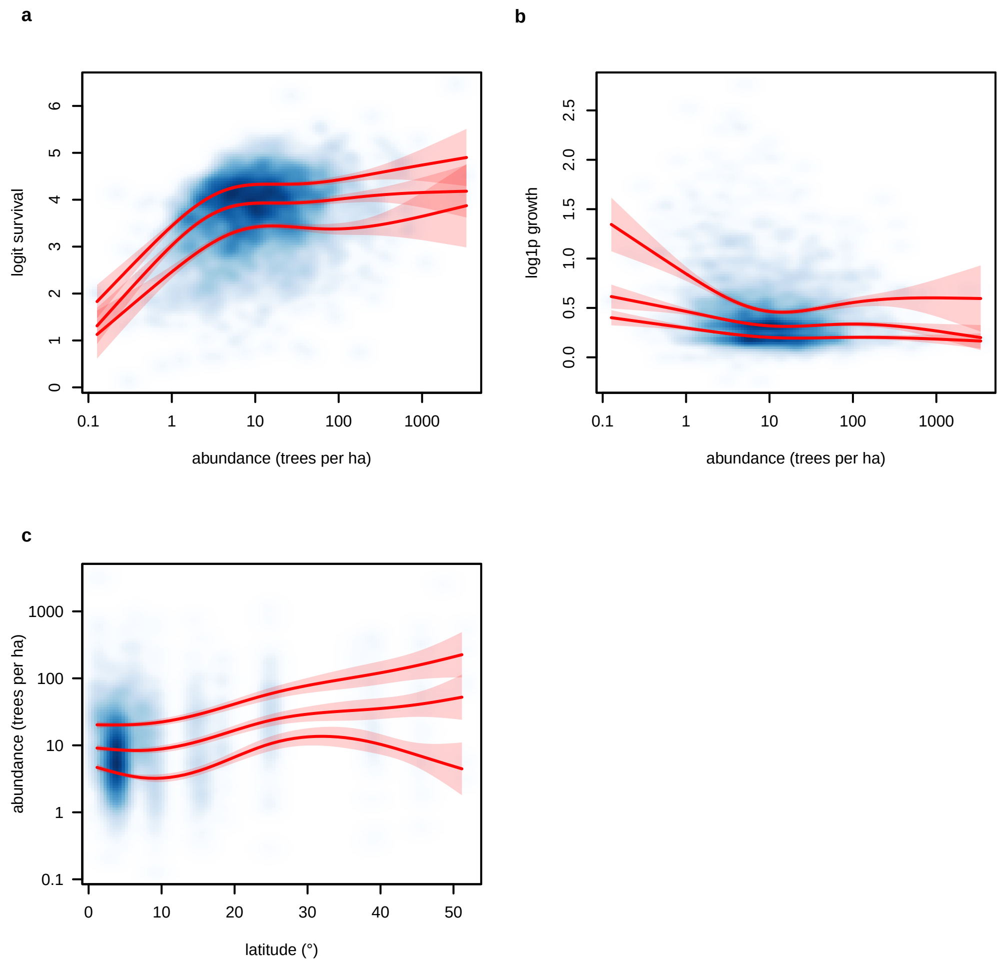

Supplementary Fig. 1 | Species overview and data coverage. Species average vital rates **(a)** survival and **(b)** growth vary with species abundance. Although there are similarly rare and common species in temperate forests as tropical forests, the number of species considerably increases toward the tropics **(c)**. Species abundance was measured as the number of trees with DBH ≥1 cm per hectare. Mean annual survival rates were based on the intercept of GLMs without predictors, here shown logit-transformed. Species-specific growth was calculated as the median of the annual DBH increment, here shown log-transformed after adding 1. Smoothed densities (blue) were obtained through 2D kernel density estimation. Regression lines and 95% confidence intervals (red) were obtained from generalized additive quantile regressions with smoothing splines for the 0.25, 0.5, and 0.75 quantile in each panel.

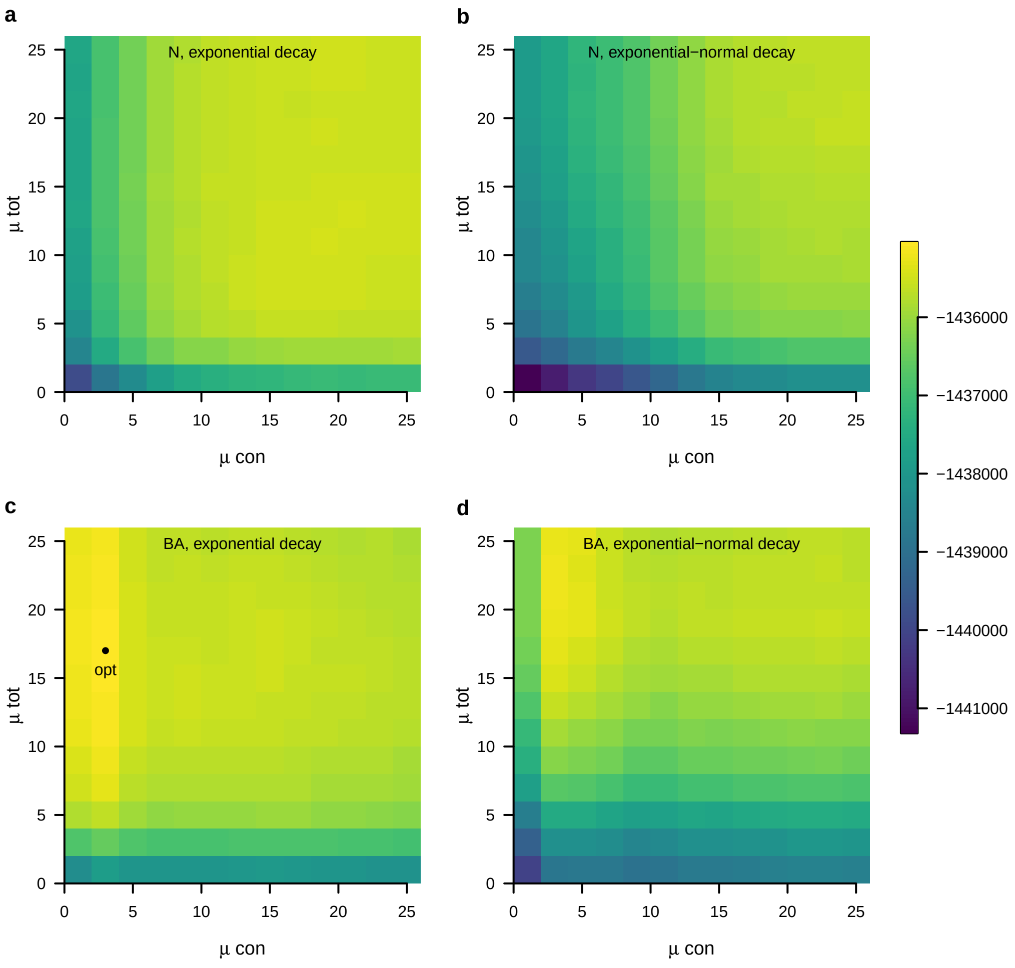

Supplementary Fig. 2 | Log-likelihood (LL) for varying definitions of local conspecific and total density in a grid search approach. The estimator for local density (i.e., N or BA), the shape of the decay function (i.e., exponential or exponential-normal) and its parameter μ were varied. The global optimum of LL was at $\mu = 3$ and 17 for conspecific and total density, respectively, with density measured as BA with an exponential distance decay.

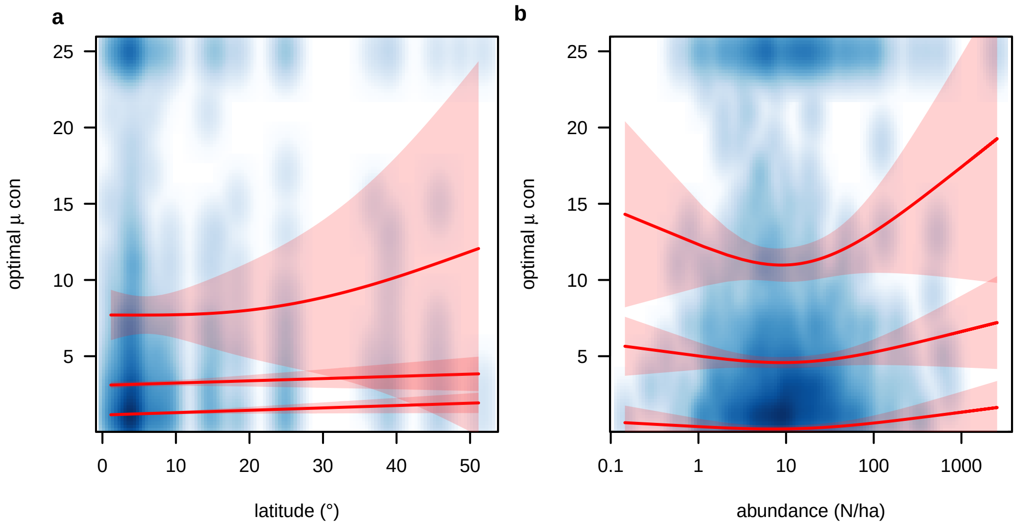

Supplementary Fig. 3 | Relationship between species-specific optima in *μ* for conspecific densities and (a) absolute latitude and (b) species abundance. Species-specific optima in *μ* are shown for those species for which the grid search yielded a distinct optimum of the log likelihood (n = 1207) and for the setting that was optimal overall, i.e., exponential distance decay and densities measured as basal area. Smoothed densities (blue) were obtained through 2D kernel density estimation. Regression lines and 95% confidence intervals (red) were obtained from generalized additive quantile regressions with smoothing splines for the 0.25, 0.5, and 0.75 quantile in each panel.

Supplementary tables

Supplementary Table 1 | Characteristics of the forest sites from the ForestGEO network used in this study. MAT and MAP are mean annual temperature and precipitation, respectively. Plot size refers to the area of a plot that was used in this study.

Site	Latitude (°)	Longitude (°)	MAT (°C)	MAP (mm/yr)	Plot size (ha)	N censuses	Census years
Amacayacu	-3.81	-70.27	25.8	3,216	25.0	2	2007, 2014
Barro Colorado Island	9.15	-79.85	27.1	2,551	50.0	8	1981, 1985, 1990, 1995, 2000, 2005, 2010, 2015
Edoro - Ituri	1.44	28.58	24.3	1,682	20.0	3	1996, 2001, 2007
Fushan	24.76	121.56	18.2	4,271	25.0	4	2004, 2008, 2013, 2018
Huai Kha Khaeng	15.63	99.22	23.5	1,476	50.0	4	1994, 1999, 2004, 2009
Ilha do Cardoso	-25.10	-47.96	22.4	2,100	10.2	2	2009, 2019
Khao Chong	7.54	99.80	27.1	2,611	24.0	3	2000, 2005, 2010
Korup	5.07	8.85	26.6	5,272	50.0	2	1998, 2008
La Planada	1.16	-77.99	19.0	4,087	25.0	2	1997, 2003
Lambir	4.19	114.02	26.6	2,664	52.0	4	1992, 1997, 2003, 2008
Lenda - Ituri	1.44	28.58	24.3	1,682	20.0	3	1995, 2001, 2007
Lilly Dickey Woods	39.24	-86.22	11.6	1,203	25.0	2	2012, 2017
Luquillo	18.33	-65.82	22.8	3,548	16.0	4	2001, 2006, 2011, 2016
Mo Singto	14.43	101.35	23.5	2,100	30.0	3	2004, 2010, 2016
Pasoh	2.98	102.31	27.9	1,788	50.0	6	1987, 1990, 1995, 2000, 2005, 2010
Santa Cruz	37.01	-122.08	14.8	778	6.0	3	2007, 2012, 2018
Sinharaja	6.40	80.40	22.5	5,016	25.0	3	1995, 2001, 2007
Smithsonian Conservation Biology Institute	38.89	-78.15	12.9	1,001	25.6	2	2008, 2013
Smithsonian Environmental Research Center	38.89	-76.56	13.2	1,068	16.0	2	2008, 2014
Wabikon	45.55	-88.79	4.2	805	25.6	3	2008, 2013, 2018
Wind River	45.82	-121.96	9.2	2,495	27.2	2	2011, 2016
Wytham Woods	51.77	-1.34	10.0	717	18.0	4	2008, 2010, 2016, 2021
Zofin	48.66	14.71	6.2	866	25.0	2	2012, 2017

Supplementary notes

Amacayacu

The 25-ha Long-Term Ecological Research Project of Amacayacu is a collaborative project of the Instituto Amazónico de Investigaciones Científicas Sinchi and the Universidad Nacional de Colombia Sede Medellín, in partnership with the Unidad de Manejo Especial de Parques Naturales Nacionales and the Forest Global Earth Observatory of the Smithsonian Tropical Research Institute (ForestGEO). The Amacayacu Forest Dynamics Plot is part of ForestGEO, a global network of large-scale demographic tree plots. We acknowledge the Director and staff of the Amacayacu National Park for supporting and maintaining the project in this National Park.

Barro Colorado Island

The BCI forest dynamics research project was made possible by National Science Foundation grants to Stephen P. Hubbell: DEB-0640386, DEB-0425651, DEB-0346488, DEB-0129874, DEB-00753102, DEB-9909347, DEB-9615226, DEB-9615226, DEB-9405933, DEB-9221033, DEB-9100058, DEB-8906869, DEB-8605042, DEB-8206992, DEB-7922197, support from the Forest Global Earth Observatory, the Smithsonian Tropical Research Institute, the John D. and Catherine T. MacArthur Foundation, the Mellon Foundation, the Small World Institute Fund, and numerous private individuals, and through the hard work of over 100 people from 10 countries over the past three decades. The plot project is part of the Forest Global Earth Observatory (ForestGEO), a global network of large-scale demographic tree plots.

References

Condit R., Perez, R., Aguilar, S., Lao, S., Foster, R., Hubbell, S.P. 2019. Complete data from the Barro Colorado 50-ha plot: 423617 trees, 35 years, 2019 version.

Condit, R. 1998. Tropical Forest Census Plots. Springer-Verlag and R. G. Landes Company, Berlin, Germany, and Georgetown, Texas.

Hubbell, S.P., R.B. Foster, S.T. O'Brien, K.E. Harms, R. Condit, B. Wechsler, S.J. Wright, and S. Loo de Lao. 1999. Light gap disturbances, recruitment limitation, and tree diversity in a neotropical forest. Science 283: 554-55^7.

Condit R., Perez, R., Aguilar, S., Lao, S., Foster, R., Hubbell, S.P. 2019. BCI 50-ha plot taxonomy, 2019 version.

Fushan

The Fushan Forest Dynamics plot (FDP) is supported by the Taiwan Forestry Bureau, the Taiwan Forestry Research Institute and the Ministry of Science and Technology of Taiwan. We would like to express our gratitude to all field technicians and students who helped with the implementation and recensus of the Fushan FDP. We also thank the Fushan Research Center staff for providing logistic support.

Huai Kha Khaeng

The Huai Kha Khaeng 50-hectare plot project has been financially and administratively supported by many institutions and agencies. Direct financial support for the plot has been provided by the Royal Thai Forest Department and the National Parks Wildlife and Plant Conservation Department, the Arnold Arboretum of Harvard University (under NSF award #DEB-0075334, and grants from USAID and the Rockefeller Foundation), the Smithsonian Tropical Research Institute, and the National Institute for Environmental Studies, Japan. The Huai Kha Khaeng Forest Dynamics Plot is part the Forest Global Earth Observatory (ForestGEO), a global network of large-scale demographic tree plots. We acknowledge the Royal Thai Forest Department for supporting and maintaining the project in Huai Kha Khaeng Wildlife Sanctuary, Thailand.

References

Bunyavejchewin, S., LaFrankie, J. V., Baker, P. J., Davies, S. J., and Ashton, P. S. 2009. Forest trees of Huai Kha Khaeng Wildlife Sanctuarry, Thailand: Data from the 50-hectare Forest Dynamic Plot. The National Parks, Wildlife and Plant Conservation Department.

Bunyavejchewin, S., Baker, P. J., LaFrankie, J. V., and Ashton, P. S. 2001. Stand structure of a seasonal dry evergreen forest at Huai Kha Khaeng Wildlife Sanctuary, western Thailand. Natural History Bulletin of the Siam Society, 49: 89-106.

Bunyavejchewin, S., LaFrankie, J. V., Pattapong, P., Kanzaki, M., Itoh, A., Yamakura, T., and Ashton, P. S. 1998. Topographic analysis of a large-scale research plot in seasonal dry evergreen forest at Huai Kha Khaeng Wildlife Sanctuary, Thailand. Tropics, 8: 45-60.

Ilha do Cardoso

The 10-ha Ilha do Cardoso Forest Dynamics Plot was established with support from the project Parcelas Permanente São Paulo (PPSP, BIOTA-FAPESP) and the Universidade de São Paulo. Thank you to the plot Principal Investigator Alexandre Adalardo de Oliveira and many field workers, research and data technicians, and staff.

Ituri - Edoro and Lenda

The Ituri 40-ha plot program is a collaborative project between the Centre de Formation et de Recherche en Conservation Forestière, the Wildlife Conservation Society – DRC through his conservation project in the Okapi forest Reserve, in partnership with the Forest Global Earth Observatory (ForestGEO). The Ituri plots are financially supported by the Wildlife Conservation Society, the Frank Levinson Family Foundation, and ForestGEO. The Institut Congolais pour la Conservation de la Nature graciously provided the research permit.

References

Makana J-R., Hart T.B., Liengola I., Ewango C.N.E., Hart J.A. & Condit R. (2004). Ituri Forest Dynamics Plots, Democratic Republic of Congo. In Losos E.C. and Leigh, E.G. Jr. eds. Forest Diversity and Dynamism: Findings from a Large-Scale Plot Network. . 506-516. University of Chicago Press, Chicago.

Khao Chong

The 24-hectare Khao Chong Forest Dynamics Plot is a collaborative project of the Royal Thai Forest Department, the Department of National Parks Wildlife and Plant Conservation, Thailand, the Arnold Arboretum of Harvard University, the Smithsonian Tropical Research Institute. The Khao Chong Forest Dynamics Plot is part of the Center for Tropical Forest Science, a global network of large-scale demographic tree plots. We acknowledge the Department of National Parks Wildlife and Plant Conservation, Thailand for supporting and maintaining the project in Khao Ban Tad Wildlife Sanctuary, Thailand.

Korup

The 50-ha is a collaborative project of the University of Buea, Cameroon, and the World Wide Fund for Nature, Cameroon Program in partnership with the Forest Global Earth Observatory of the Smithsonian Tropical Research Institute (ForestGEO). Funding for the first census was provided by the International Cooperative Biodiversity Group (a consortium

of the NIH, the NSF, and the USDA), with supplemental funding by the Central Africa Regional Program for the Environment (a program of USAID). Funding for the second census was provided by the Frank Levinson Family Foundation. Permission to conduct the field program in Cameroon is provided by the Ministry of Environment and Forests and the Ministry of Scientific Research and Innovation.

References

Chuyong G.B., Condit, R., Kenfack, D., Losos, E., Sainge, M., Songwe, N.C., and Thomas, D.W. (2004). Korup Forest Dynamics Plot, Cameroon. In Losos E.C. and Leigh, E.G. Jr. eds. Forest Diversity and Dynamism: Findings from a Large-Scale Plot Network. . 506-516. University of Chicago Press, Chicago.

Thomas, D.W., Kenfack, D., Chuyong, G.B., Sainge N.M., Losos E.C., Condit R.S., Songwe N.C. (2003). Tree Species of Southwestern Cameroon: Tree distribution maps, diameter tables and species documentation of the 50-ha Korup Forest Dynamics Plot. Center for Tropical Forest Science, Washington, D.C.

Kenfack D., Thomas D.W., Chuyong G.B., & Condit R. (2007). Rarity and abundance in a diverse African forest: Biodiversity Conservation 16: 2045 – 2074.

Lambir

The 52-ha Long-Term Ecological Research Project is a collaborative project of the Forest Department of Sarawak, Malaysia, the Forest Global Earth Observatory (ForestGEO) , the Arnold Arboretum of Harvard University, USA (under NSF awards DEB-9107247 and DEB-9629601), and Osaka City, Ehime & Kyoto Universities, Japan (under MEXT/JSPS KAKENHI grants 09NP0901, 22H02388, and JST/JICA-SATREPS PUBS). The Lambir Forest Dynamics Plot is part of ForestGEO, a global network of large-scale demographic tree plots. We acknowledge the Sarawak Forest Department for supporting and maintaining the project in Lambir Hills National Park.

References

Lee, H.S., P.S. Ashton, T. Yamakura, S. Tan, S.J. Davies, A. Itoh, T. Ohkubo & J.V. LaFrankie. (2002). The 52-Hectare Forest Dynamics Plot at Lambir Hills, Sarawak, Malaysia: Tree Distribution Maps, Diameter Tables and Species Documentation.Sarawak Forest Department. 621 pp. Lee Ming Press, Kuching, Sarawak, Malaysia.

Lee, H., Davies, S. J., LaFrankie, J. V., Tan, S., Itoh, A., Yamakura, T., and Ashton, P. S. 2002. Floristic and structural diversity of 52 hectares of mixed dipterocarp forest in Lambir Hills National Park, Sarawak, Malaysia. Journal of Tropical Forest Science, 14: 379-400.

La Planada

The 25-ha is a collaborative project between the Instituto de Investigación de Recursos Biológicos Alexander von Humboldt and the Forest Global Earth Observatory (ForestGEO) of the Smithsonian Tropical Research Institute. We especially thank Martha Isabel Vallejo and Cristian Samper, who made this project possible. For more information on La Planada, visit: http://i2d.humboldt.org.co/ceiba/resource.do?r=planada_parcelapermanente_censo1

Lilly Dickey Woods

The 25-ha Indiana University Forest Dynamics Plot is a collaborative project of Indiana University and the Center for Tropical Forest Science of the Smithsonian Tropical Research Institute. Funding for the installation and maintenance of the IUFDP came from multiple sources including Indiana Academy of Science, Indiana University Research and Teaching Preserve, U.S. Department of Energy, the USDA National Institute for Food and Agriculture McIntire Stennis project 1018790, and ForestGEO. The IUFDP is part of ForestGEO, a global network of large-scale demographic tree plots.

Reference

Johnson, D.J., Clay, K. & Phillips, R.P. Mycorrhizal associations and the spatial structure of an old-growth forest community. Oecologia 186, 195–204 (2018). https://doi.org/10.1007/s00442-017-3987-0

Luquillo

This research was supported by grants BSR-8811902, DEB 9411973, DEB 0080538, DEB 0218039, DEB 0620910, DEB 0963447 AND DEB-129764 from NSF to the Department of Environmental Science, University of Puerto Rico, and to the International Institute of Tropical Forestry, USDA Forest Service, as part of the Luquillo Long-Term Ecological Research Program. The U.S. Forest Service (Dept. of Agriculture) and the University of Puerto Rico gave additional support. The LFDP is part of the Smithsonian Institution Forest Global Earth Observatory, a worldwide network of large, long-term forest dynamics plots.

Reference

Zimmerman, Jess K., Liza S. Comita, Jill Thompson, María Uriarte, and Nicholas Brokaw. 2010. Patch dynamics and community metastability of a subtropical forest: compound effects of natural disturbance and human land use. Landscape Ecology 25: 1099-1111.

Mo Singto

The 30.5-ha Mo Singto Forest Dynamics Plot is supported by Mahidol University, National Center for Genetic Engineering and Biotechnology, National Science and Technology Development Agency, and Thai Ministry of Natural Resources and Environment. Many thanks to plot Principal Investigators Anuttara Nathalang and Warren Y. Brockelman, and to countless field workers, research and data technicians, and staff.

Reference

Brockelman WY, Nathalang A, Maxwell JF (eds.), 2017. Mo Singto Forest Dynamics Plot: Flora and Ecology, National Science and Technology Development Agency, and Department of National Parks, Wildlife and Plant Conservation, Bangkok.

Pasoh

Data from the Pasoh Research Forest was provided by the Forest Research Institute Malaysia - Forest Global Earth Observatory, Smithsonian Tropical Research Institute collaborative research project. Negeri Sembilan Forestry Department is the custodian of Pasoh Research Forest and we acknowledge the department for preserving the research forest.

References

Manokaran N & LaFrankie JV. 1990. Stand structure of Pasoh Forest Reserve, A lowland rainforest in Peninsular Malaysia. Journal of Tropical Forest Science 3: 14-24.

Kochumen KM, LaFrankie JV, Manokaran N. 1990. Floristic composition of Pasoh Forest Reserve, a lowland forest in Peninsular Malaysia. Journal of Tropical Forest Science 3: 1-13.

Manokaran N, Abd Rahman K, Azman H, Quah ES, Chong PF. 1992. Short-term population dynamics of dipterocarp trees in a lowland rainforest in Peninsular Malaysia. Journal of Tropical Forest Science 5: 97-112.

Smithsonian Conservation Biology Institute - SCBI

Funding for the Smithsonian Conservation Biology Institute (SCBI) Large Forest Dynamics Plot (LFDP) was provided by the Smithsonian Institution, the National Zoological Park, and the HSBC Climate Partnership. The SCBI LFDP is part of the Smithsonian Institution Forest Global Earth Observatory, a worldwide network of large, long-term forest dynamics plots.

Reference

Bourg, N.A., W.J. McShea, J.R. Thompson, J.C. McGarvey, and X. Shen. 2013. Initial census, woody seedling, seed rain, and stand structure data for the SCBI SIGEO Large Forest Dynamics Plot. Ecology 94(9): 2111-2112. http://dx.doi.org/10.1890/13-0010.1

Smithsonian Environmental Research Center - SERC

Data on SERC Dynamic Forest plot was provided by Geoffrey Parker on October 8, 2020. These data were gathered as part of forest ecology studies at the Smithsonian Environmental Research Center (SERC). SERC is a participant in the Smithsonian Institution Forest Global Earth Observatory (ForestGEO) network.

Sinharaja

The 25-ha Long-Term Ecological Research Project at Sinharaja World Heritage Site is a collaborative project of the Uva Wellassa University, University of Peradeniya, the Forest Global Earth Observatory (ForestGEO) of the Smithsonian Tropical Research Institute, with supplementary funding received from the John D. and Catherine T. Macarthur Foundation, the National Institute for Environmental Science, Japan, and the Helmholtz Centre for Environmental Research-UFZ, Germany, for past censuses. The PIs gratefully acknowledge the Forest Department, Uva Wellassa University, and the Post-Graduate Institute of Science at the University of Peradeniya, Sri Lanka for supporting this project, and the local field and lab staff who tirelessly contributed in the repeated censuses of this plot.

University of California Santa Cruz - UCSC

The UCSC Forest Ecology Research Plot was made possible by National Science Foundation grants to Gregory S. Gilbert (DEB-0515520, DEB-084259, and DEB-1655896), by the Pepper-Giberson Chair Fund, the Robert Headley Presidential Chair for Integral Ecology and Environmental Justice, the UCSC Campus Natural Reserve, the University of California, the ForestGEO global network, and by the hard work of hundreds of UCSC

students. We acknowledge that the land on which this research was conducted is the unceded territory of the Awaswas-speaking Uypi Tribe. The Amah Mutsun Tribal Band, comprised of the descendants of indigenous people taken to missions Santa Cruz and San Juan Bautista during Spanish colonization of the Central Coast, is today working hard to restore traditional stewardship practices on these lands and heal from historical trauma.

Reference

Gilbert, G.S., E. Howard, B. Ayala-Orozco, M. Bonilla-Moheno, J. Cummings, S. Langridge, I.M. Parker, J. Pasari, D. Schweizer, S. Swope. 2010. Beyond the tropics: forest structure in a temperate forest mapped plot. Journal of Vegetation Science 21: 388-405.

Wabikon

The Wabikon Lake Forest Dynamics Plot, located in the Chequamegon-Nicolet National Forest of northern Wisconsin, is part of the Smithsonian Institution's ForestGEO network. Tree censuses at the site have been supported by the 1923 Fund, the Smithsonian Tropical Research Institute, and the Cofrin Center for Biodiversity at the University of Wisconsin-Green Bay. More than 50 scientists and student assistants contributed to the first two plot censuses. We are particularly grateful for the leadership of Gary Fewless, Steve Dhein, Kathryn Corio, Juniper Sundance, Cindy Burtley, Curt Rollman, Mike Stiefvater, Kim McKeefry, and U.S. Forest Service collaborators Linda Parker and Steve Janke.

Wind River

The Wind River Forest Dynamics Plot is a collaborative project of Utah State University, the Utah Agricultural Experiment Station and the USDA Forest Service Pacific Northwest Research Station. Funding was provided by the Center for Tropical Forest Science of the Smithsonian Tropical Research Institute, Utah State University, and the Utah State Agricultural Experiment Station. We acknowledge the Gifford Pinchot National Forest and the Wind River Field Station for providing logistical support, and the students, volunteers and staff individually listed at http://wfdp.org for data collection.

References

Lutz, J. A, A. J. Larson, J. A. Freund, M. E. Swanson, and K. J. Bible. 2013. The ecological importance of large-diameter trees to forest structural heterogeneity. PLOS ONE 8(12): e82784.

Lutz, J. A., A. J. Larson, T. J. Furniss, J. A. Freund, M. E. Swanson, D. C. Donato, K. J. Bible, J. Chen, and J. F. Franklin. 2014. Spatially non-random tree mortality and ingrowth maintain equilibrium pattern in an old-growth *Pseudotsuga-Tsuga* forest. *Ecology* 95(8): 2047-2054.

Wytham Woods

The 18-ha Long-Term Forest Monitoring Plot is a collaborative project between the University of Oxford, the Centre for Ecology and Hydrology, and the Smithsonian Institution ForestGEO (HSBC Climate Partnership). The Wytham Forest Monitoring Plot is part of ForestGEO, a global network of large-scale demographic tree plots. Censuses were funded with support from ForestGEO and Advanced Investigator award from European Research Council to YM (GEM-TRAIT).

Reference

Butt, N., Campbell, G., Malhi, Y., Morecroft, M., Fenn, K., Thomas, M. (2009) Initial results from establishment of a long-term broadleaf monitoring plot at Wytham Woods, Oxford, UK. University of Oxford Report.

Zofin

The Zofin Forest Dynamics Plot was established with the support of the Smithsonian Institution as a part of the Smithsonian Institution Forest Global Earth Observatory, a worldwide network of large, long-term forest dynamics plots. We acknowledge the Department of Forest Ecology of the Silva Tarouca Research Institute for supporting and maintaining the long-term monitoring of the Zofin Forest Dynamics Plot (under the Czech Science Foundation, grant No. 20-17282S)

References

Krůček M., Král K., Cushman K., Missarov A., and Kellner JR. 2020. Supervised Segmentation of Ultra-High-Density Drone Lidar for Large-Area Mapping of Individual Trees. Remote Sensing. 2020; 12(19): 3260.

Kašpar, J., Tumajer, J., Šamonil, P., and Vašíčková, I. 2021. Species-specific climate–growth interactions determine tree species dynamics in mixed Central European mountain forests. Environmental Research Letters, 16(3), 034039.

Šamonil P., Daněk P., Lutz J.A., Anderson-Teixeira, K.J., Jaroš J., Phillips J.D., Rousová A., Adam D., Larson A.J., Kašpar J., Janík D., Vašíčková I., Gonzáles-Akre E., and Egli M. 2022. Tree Mortality may Drive Landscape Formation: Comparative Study from Ten Temperate Forests. Ecosystems. 10.1007/s10021-022-00755-8.
