# Supplementary material for: Latitudinal patterns in stabilizing density dependence of forest communities
Source: Nature. 2024 Feb 28;627(8004):564–71. doi: 10.1038/s41586-024-07118-4 (PMC10954553; doi:10.1038/s41586-024-07118-4)
Supplement: Supplementary file 2 — Reporting Summary [file 41586_2024_7118_MOESM2_ESM.pdf]

nature portfolio

Corresponding author(s): Lisa Hülsmann

Last updated by author(s): Sep 19, 2023

Reporting Summary

Nature Portfolio wishes to improve the reproducibility of the work that we publish. This form provides structure for consistency and transparency in reporting. For further information on Nature Portfolio policies, see our Editorial Policies and the Editorial Policy Checklist.

Statistics

For all statistical analyses, confirm that the following items are present in the figure legend, table legend, main text, or Methods section.

n/a	Confirmed	
☐	☒	The exact sample size (*n*) for each experimental group/condition, given as a discrete number and unit of measurement
☐	☒	A statement on whether measurements were taken from distinct samples or whether the same sample was measured repeatedly
☐	☒	The statistical test(s) used AND whether they are one- or two-sided<br>*Only common tests should be described solely by name; describe more complex techniques in the Methods section.*
☐	☒	A description of all covariates tested
☐	☒	A description of any assumptions or corrections, such as tests of normality and adjustment for multiple comparisons
☐	☒	A full description of the statistical parameters including central tendency (e.g. means) or other basic estimates (e.g. regression coefficient) AND variation (e.g. standard deviation) or associated estimates of uncertainty (e.g. confidence intervals)
☐	☒	For null hypothesis testing, the test statistic (e.g. *F*, *t*, *r*) with confidence intervals, effect sizes, degrees of freedom and *P* value noted<br>*Give P values as exact values whenever suitable.*
☒	☐	For Bayesian analysis, information on the choice of priors and Markov chain Monte Carlo settings
☐	☒	For hierarchical and complex designs, identification of the appropriate level for tests and full reporting of outcomes
☐	☒	Estimates of effect sizes (e.g. Cohen's *d*, Pearson's *r*), indicating how they were calculated

Our web collection on statistics for biologists contains articles on many of the points above.

Software and code

Policy information about availability of computer code

Data collection	No software was used for data collection.
Data analysis	The analyses were conducted in R version 4.2.1 using the packages mgcv, metafor, and DHARMa. Additional packages were used for data management and visualization (see code at https://github.com/LisaHuelsmann/latitudinalCNDD).

For manuscripts utilizing custom algorithms or software that are central to the research but not yet described in published literature, software must be made available to editors and reviewers. We strongly encourage code deposition in a community repository (e.g. GitHub). See the Nature Portfolio guidelines for submitting code & software for further information.

Data

Policy information about availability of data

All manuscripts must include a data availability statement. This statement should provide the following information, where applicable:

- Accession codes, unique identifiers, or web links for publicly available datasets
- A description of any restrictions on data availability
- For clinical datasets or third party data, please ensure that the statement adheres to our policy

The forest data that support the findings of this study are available from the ForestGEO network. For some of the sites, the data is publicly available at https://forestgeo.si.edu/explore-data. Restrictions apply, however, to the availability of the data from other sites, which were used under license for the current study, and

so are not publicly available. Raw data are available from the authors upon reasonable request and with permission of the principal investigators of the ForestGEO sites. Species-site-specific CNDD estimates to reproduce the meta-analyses are available at https://github.com/LisaHuelsmann/latitudinalCNDD.

Human research participants

Policy information about studies involving human research participants and Sex and Gender in Research.

Reporting on sex and gender	does not apply - I think this part should have been hidden
Population characteristics	*Describe the covariate-relevant population characteristics of the human research participants (e.g. age, genotypic information, past and current diagnosis and treatment categories). If you filled out the behavioural & social sciences study design questions and have nothing to add here, write "See above."*
Recruitment	*Describe how participants were recruited. Outline any potential self-selection bias or other biases that may be present and how these are likely to impact results.*
Ethics oversight	*Identify the organization(s) that approved the study protocol.*

Note that full information on the approval of the study protocol must also be provided in the manuscript.

Field-specific reporting

Please select the one below that is the best fit for your research. If you are not sure, read the appropriate sections before making your selection.

☐ Life sciences ☐ Behavioural & social sciences ☒ Ecological, evolutionary & environmental sciences

For a reference copy of the document with all sections, see nature.com/documents/nr-reporting-summary-flat.pdf

Ecological, evolutionary & environmental sciences study design

All studies must disclose on these points even when the disclosure is negative.

Study description	We used repeated census data from twenty-three large forest sites around the globe to analyze latitudinal patterns in conspecific negative density dependence (CNDD) following a three-step approach: We fitted species-site-specific mortality models from repeated observations of individual trees using neighborhood densities as a predictor. Then, we used these models to quantify CNDD for each species and site using an estimator designed to maximize robustness, comparability, and relevance for fitness and stabilization. Finally, we used meta-regressions to explore latitudinal patterns in CNDD. Robustness of the analysis pipeline was validated by model diagnostics and randomization.
Research sample	The data used in this study were collected at twenty-three forest sites with permanent forest dynamics plots that are part of the Forest Global Earth Observatory network (ForestGEO).
Sampling strategy	All free-standing woody stems with diameter ≥1 cm at 1.3 m from the ground (DBH) are censused at each site, with between 9,718 and 495,577 mapped tree individuals at each site. For the mortality analyses, we selected observations of all alive trees of non-fern and non-palm species with a DBH < 10 cm in one census and follow-up data in a consecutive census.
Data collection	The census data collected for each individual stem or tree include species, DBH, spatial coordinates and status (alive or dead).
Timing and spatial scale	The plots vary in size between 6 and 52 ha. At all sites included in this study, two or more censuses have been carried out with remeasurement intervals of approximately five years. Censuses were carried out between 1981 and 2021.
Data exclusions	Mortality analyses were restricted to small trees (between 1 and 10 cm DBH) because it is assumed that CNDD effects are most pronounced in early life stages. Observations of trees or stems were excluded when information on coordinates, species, status, or date of measurement was missing.
Reproducibility	This study is based on observational data, and no experiments were conducted.
Randomization	This study is based on observational data. Therefore, randomization into groups does not apply.
Blinding	Blinding is not relevant for this study because the data were not specifically collected to assess density dependence.

Did the study involve field work? ☐ Yes ☒ No

Reporting for specific materials, systems and methods

We require information from authors about some types of materials, experimental systems and methods used in many studies. Here, indicate whether each material, system or method listed is relevant to your study. If you are not sure if a list item applies to your research, read the appropriate section before selecting a response.

Materials & experimental systems

n/a	Involved in the study	
☒	☐	Antibodies
☒	☐	Eukaryotic cell lines
☒	☐	Palaeontology and archaeology
☒	☐	Animals and other organisms
☒	☐	Clinical data
☒	☐	Dual use research of concern

Methods

n/a	Involved in the study	
☒	☐	ChIP-seq
☒	☐	Flow cytometry
☒	☐	MRI-based neuroimaging
